# Approximating complex musculoskeletal biomechanics using multidimensional autogenerating polynomials

**DOI:** 10.1371/journal.pcbi.1008350

**Published:** 2020-12-16

**Authors:** Anton Sobinov, Matthew T. Boots, Valeriya Gritsenko, Lee E. Fisher, Robert A. Gaunt, Sergiy Yakovenko

**Affiliations:** 1 Rockefeller Neuroscience Institute, School of Medicine, West Virginia University, Morgantown, WV, United States of America; 2 Department of Neuroscience, School of Medicine, West Virginia University, Morgantown, West Virginia, United States of America; 3 Department of Mechanical and Aerospace Engineering, Benjamin M. Statler College of Engineering and Mineral Resources, West Virginia University, Morgantown, WV, United States of America; 4 Department of Human Performance, School of Medicine, West Virginia University, Morgantown, WV, United States of America; 5 Department of Physical Medicine and Rehabilitation, University of Pittsburgh, Pittsburgh, PA, United States of America; 6 Department of Bioengineering, University of Pittsburgh, Pittsburgh, PA, United States of America; 7 Center for the Neural Basis of Cognition, Pittsburgh, PA, United States of America; University of Cincinnati, UNITED STATES

## Abstract

Computational models of the musculoskeletal system are scientific tools used to study human movement, quantify the effects of injury and disease, plan surgical interventions, or control realistic high-dimensional articulated prosthetic limbs. If the models are sufficiently accurate, they may embed complex relationships within the sensorimotor system. These potential benefits are limited by the challenge of implementing fast and accurate musculoskeletal computations. A typical hand muscle spans over 3 degrees of freedom (DOF), wrapping over complex geometrical constraints that change its moment arms and lead to complex posture-dependent variation in torque generation. Here, we report a method to accurately and efficiently calculate musculotendon length and moment arms across all physiological postures of the forearm muscles that actuate the hand and wrist. Then, we use this model to test the hypothesis that the functional similarities of muscle actions are embedded in muscle structure. The posture dependent muscle geometry, moment arms and lengths of modeled muscles were captured using autogenerating polynomials that expanded their optimal selection of terms using information measurements. The iterative process approximated 33 musculotendon actuators, each spanning up to 6 DOFs in an 18 DOF model of the human arm and hand, defined over the full physiological range of motion. Using these polynomials, the entire forearm anatomy could be computed in <10 μs, which is far better than what is required for real-time performance, and with low errors in moment arms (below 5%) and lengths (below 0.4%). Moreover, we demonstrate that the number of elements in these autogenerating polynomials does not increase exponentially with increasing muscle complexity; complexity increases linearly instead. Dimensionality reduction using the polynomial terms alone resulted in clusters comprised of muscles with similar functions, indicating the high accuracy of approximating models. We propose that this novel method of describing musculoskeletal biomechanics might further improve the applications of detailed and scalable models to describe human movement.

## Author summary

The community in the fields of biomechanics, neural engineering, and neuroscience has the need to understand and simulate realistic muscle actions in real-time. In biomechanics, the models of muscle structure have been of paramount importance for understanding the mechanical demands of movements. In neural engineering, the use of biomimetic control schemes require realistic and real-time computations with low latencies to achieve an intuitive interface with high-dimensional active prostheses or orthoses. In neuroscience, the new realization of the close relationship between neural computations and body mechanics has been promoted under the concept of neuromechanics. This concept has been instrumental in the understanding of neural computations for movement planning and execution. To enable the theoretical framework of neuromechanical computations embedded within musculoskeletal organization we propose a novel method for calculating muscle biomechanics in real-time with objective approximations that embed structural and functional attributes of simulated muscles. This description offers a scalable solution that accurately computes muscle kinematic states with real-time latencies surpassing the previous results by an order of magnitude.

## Introduction

The remarkable dexterity of the hand results from the coordinated motion of 27 kinematic degrees of freedom (DOF) actuated by arm and hand muscles. This complex coordination problem is solved continuously by our neuromuscular system without perceived cognitive effort. Yet, for prosthetic applications, the current approaches, such as pattern recognition and mode switching require significant training time [1]. Moreover, the skill and cognitive load required for continuous prosthetic control increases with the number of available prosthetic DOFs [2]. This phenomenon is captured by *the dimensionality curse* problem in movement planning, which occurs due to the increasing volume of possible solutions with the increasing number of dimensions. Recently, machine learning statistical methods have gained popularity in computer vision and robotic control problems of comparable complexity. In particular, deep learning algorithms are capable of remarkable performance in vision and language tasks [3] and significantly outperform more standard neural networks with just a few hidden layers. These performance gains and the resistance to the dimensionality curse are enabled by the hierarchical processing inherent in these multilayer deep networks, which is a biomimetic property common to biological cortical networks [4]. However, training these deep networks requires large amounts of labelled data and usually results in a black-box transformation, without any transparent internal mechanisms that would generate insights into the underlying control scheme [reviewed in 5]. In addition, machine learning solutions often require episodic model retraining [6], and rely on a considerable memory space to store the necessary parameters [7]. These constraints pose significant challenges for real-time control systems for both phenomenological and mechanistic models of human hand biomechanics. Overall, this approach limits our understanding of model boundaries, the reliable domain of operation, and, importantly, the principles of the modelled system that can be tested and improved further. Instead, using mechanistic alternatives based on known biology may overcome these limitations.

Transforming biological signals into intended prosthetic movements using biomimetic principles may solve the problem of integration between the biological and technological control systems. These systems may often be at odds with each other due to the discord in expected and executed movement. Thus, the challenges of biomimetic approaches are in specifying and implementing valid motor control theories. One such dominant theory focuses on internal models expressed within the nervous system [8–10]; it embodies an engineering concept termed the Smith predictor [11]. This theory relies on accurate estimates of the controlled plant to overcome both nonlinear dynamics and temporal delays. Another complimentary concept is *neuromechanical tuning* [12–14], which postulates reliance on the interplay between coupled neural and mechanical dynamics within the closed-loop control system. The key idea of these theories is that the description of control and its use, e.g., for prosthetics, requires an adequate description of body dynamics and musculoskeletal (MS) biomechanics [15–17]. The recent use of MS models for human-machine interfaces [18] shows promising results for this type of approach.

Musculoskeletal modelling is an important scientific tool in theoretical motor control [19–21] and its applications in human-machine interfaces [18,22]. MS models are typically comprised of geometrical descriptions of each joint’s DOF and the muscle paths around these DOFs. A muscle’s contribution to joint torque depends on the moment arm, the distance to the DOF axis of rotation, as well as muscle force, which can be described in part by muscle length and velocity that alter force generation [23,24]. Calculating these MS kinematic variables in a specific posture requires computation of the shortest path between the points of attachment in the presence of objects like bones and other muscles around which a muscle wraps [25]. Software packages like OpenSim (SimTK) provide tools for computing these kinematic variables based on anatomically accurate 3D models of the MS system. These calculations are very computationally costly and can only be performed in real-time for simple models. However, models of increasing complexity are required in both research and applications, rapidly raising their computational cost to burdensome levels.

The computational load of MS models has led to the development of multiple approximation methods that improve computational efficiency. Menegaldo and colleagues [26] proposed a series of multidimensional polynomials describing the MS variables of human leg muscles. Later these polynomials were used to simulate the musculotendon dynamics of upper [27] and lower limbs [28]. This approach supports very high computational performance with low requirements on the available memory and the number of mathematical operations. However, the generalizability of this method is limited by the hand-selected polynomial structure, which begins to have significant errors in the more complex biomechanical scenarios that occur in the hand. Addressing this limitation is not trivial as the polynomial structure itself becomes considerably more difficult as MS complexity increases. For example, muscles actuating the thumb may cross seven DOFs (three wrist and four thumb), potentially resulting in a 7-dimensional polynomial to describe its behavior. Another approach developed by Sartori and colleagues [29] emphasizes the quality of approximation using cubic splines. Albeit computationally expensive, the ability of this approach to operate at real-time has been shown in a 3-DOF per muscle model [30]. The drawback of cubic splines, however, is their limited scalability; the number of spline coefficients increases exponentially with the number of DOFs that the muscle crosses. Ultimately, both methods aim to simplify complex MS calculations, yet exhibit problems with accommodating increasing model complexity, severely limiting MS structure analysis and application.

In this study, we were driven by the rationale to develop an objective algorithm to generate phenomenological MS models capturing the input-output relationship. Then, we tested the utility of this modeling approach by testing the potential of the generated models to capture muscle functions. We present an information theory-based algorithm for approximating kinematic variables with polynomials that increase their term complexity linearly with the increasing problem complexity. This linearization of the dimensionality problem is achieved through the search for the optimal structure of approximating functions. We use spatiotemporal metrics of quality that assess approximation error and evaluate the computational time of the developed model with 33 musculotendon actuators crossing multiple DOFs each (up to 6 DOFs per muscle). Similar to other accurate phenomenological models [31], approximations can represent dexterous structural and, to a lesser extent, functional details.

## Methods

The approximation of muscle path kinematic variables consisted of three steps: *i*) creating a dataset describing muscle length and moment arm values for all physiological postures using the OpenSim model; *ii*) searching for a set of optimal polynomials approximating kinematic variables implemented with a physical constraint between muscle moment arms and muscle length; and *iii*) validating the produced polynomials.

### Dataset

**Fig 1:**
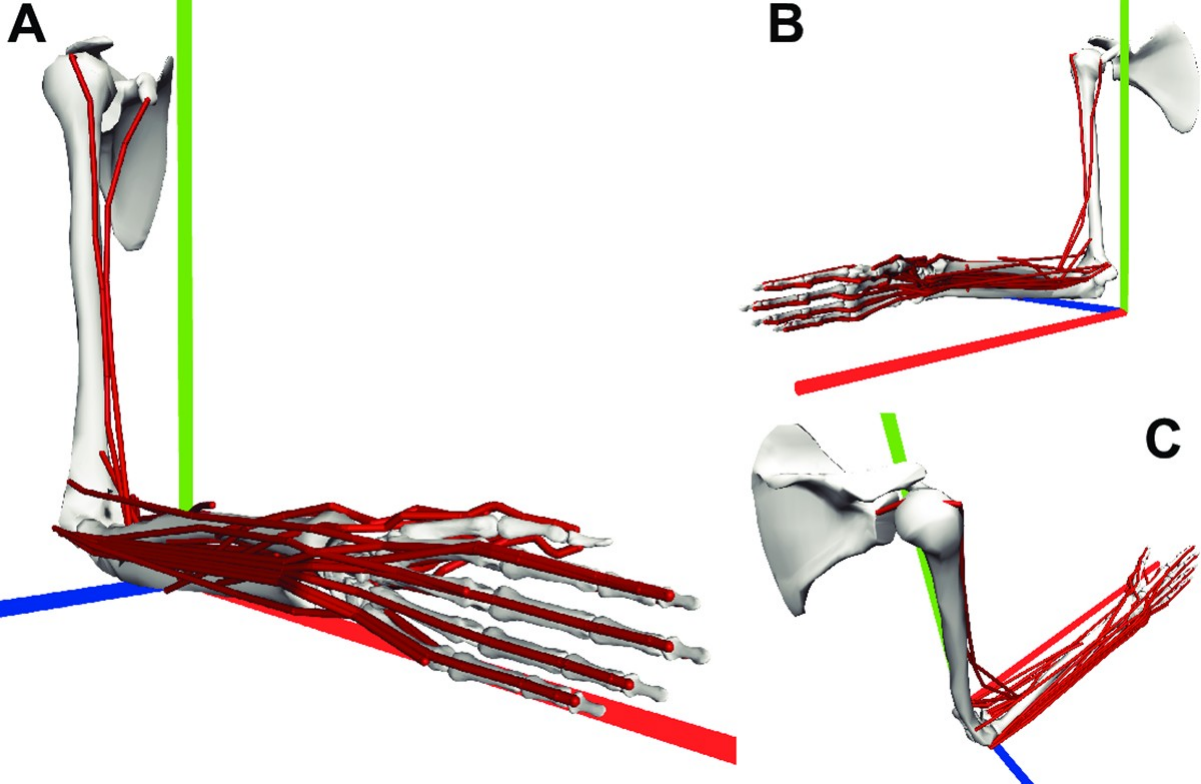
Upper-limb representation in OpenSim from three points of view. The geometry of muscle paths is shown in red for the displayed posture. Global axes: X (red), Y (green), Z (blue). **A**. Lateral view. **B**. Medial view. **C**. Top view.

We used a previously developed model of the arm and hand (Fig 1) to capture the relationship between muscle lengths and moment arms in all physiological postures [32–34]. The model contains 33 musculotendon actuators, some representing multiple heads of the same muscle, spanning 18 physiological DOFs (see Tables 1 and 2 in Appendix) and was implemented in OpenSim software [25]. Similar to the previous study of Sartori et al. [29] the values for the kinematic variables were obtained on a uniform grid with 9 points per DOF, resulting in the domain size of 9^*d*^ data points per muscle, where *d* is the number of DOFs that a muscle crosses. The extreme positions were included so that 9 points were selected within the range from 0% to 100% of DOF range. For example, since the *extensor carpi ulnaris* muscle spans two DOFs (wrist flexion-extension and pronation-supination) in our model (ulna deviation is not simulated) its moment arms and muscle lengths were sampled in 9^2^ = 81 positions. This 9-point dataset contained 674,937 points for all available postures in the model. In addition, to compare the approximations achieved with different methods (described below), we generated an 8-point dataset containing 348,136 values sampled between the values of the 9-point dataset.

**Table 1:** The notation of polynomial terms using index and the corresponding *K*-notation expressed with kinematic posture coordinates (*x*_1_, *x*_2_, *x*_3_, *x*_4_, *x*_5_).

*v*-axis index	Unique combination (power)	Examples of polynomial terms	*K*-notation of polynomial structure
1	(1)	*x*_1_, *x*_2_	*K*_*1*_, *K*_*2*_
2	(2)	$x_{1}^{2}$	*K*_*11*_
3	(3)	$x_{1}^{3}$	*K*_*111*_
4	(4)	$x_{1}^{4}$	*K*_*1111*_
5	(5)	$x_{1}^{5}$	*K*_*11111*_
6	(1,1)	*x*_1_*x*_2_, *x*_2_*x*_3_	*K*_*12*_, *K*_*23*_
7	(1,2)	$x_{1}^{2}x_{2}$, $x_{2}x_{3}^{2}$	*K*_*112*_, *K*_*233*_
8	(1,3)	$x_{1}x_{2}^{3}$	*K*_*1222*_
9	(1,4)	$x_{1}x_{2}^{4}$	*K*_*12222*_
10	(2,2)	$x_{1}^{2}x_{2}^{2}$	*K*_*1122*_
11	(2,3)	$x_{1}^{2}x_{2}^{3}$	*K*_*11222*_
12	(1,1,1)	*x*_1_*x*_2_*x*_3_	*K*_*123*_
13	(1,1,2)	$x_{1}x_{2}x_{3}^{2}$	*K*_*1233*_
14	(1,1,3)	$x_{1}x_{2}x_{3}^{3}$	*K*_*12333*_
15	(1,2,2)	$x_{1}x_{2}^{2}x_{3}^{2}$	*K*_*12233*_
16	(1,1,1,1)	*x*_1_*x*_2_*x*_3_*x*_4_	*K*_*1234*_
17	(1,1,1,2)	$x_{1}x_{2}x_{3}x_{4}^{2}$	*K*_*12344*_
18	(1,1,1,1,1)	*x*_1_*x*_2_*x*_3_*x*_4_*x*_5_	*K*_*12345*_

**Table 2:** Model performance comparison. Cubic spline (CS) and two polynomial approximations with and without the constraint linking muscle lengths and moment arms (constrained and unconstrained polynomials, CP and UP), as described by algorithm in Model Physical Constraints in Methods. L is length, MA is moment arms.

Method	RMS error ± standard deviation, %		Total number of parameters		AIC, au	
	L	MA	L	MA	L	MA
CS	1.34*10^−5^ ± 1.56*10^−5^	1.84*10^−6^ ± 2.47*10^−6^	1.1*10^9^	1.64*10^10^	2.2*10^9^	3.2*10^10^
UP	0.0383 ± 0.0918	0.757 ± 1.477	610	705	-6.7*10^6^	-5.7*10^5^
CP	0.0382 ± 0.0910	0.757 ± 1.477	661	783	-6.7*10^6^	-5.7*10^5^

### Model structure

**Fig 2:**
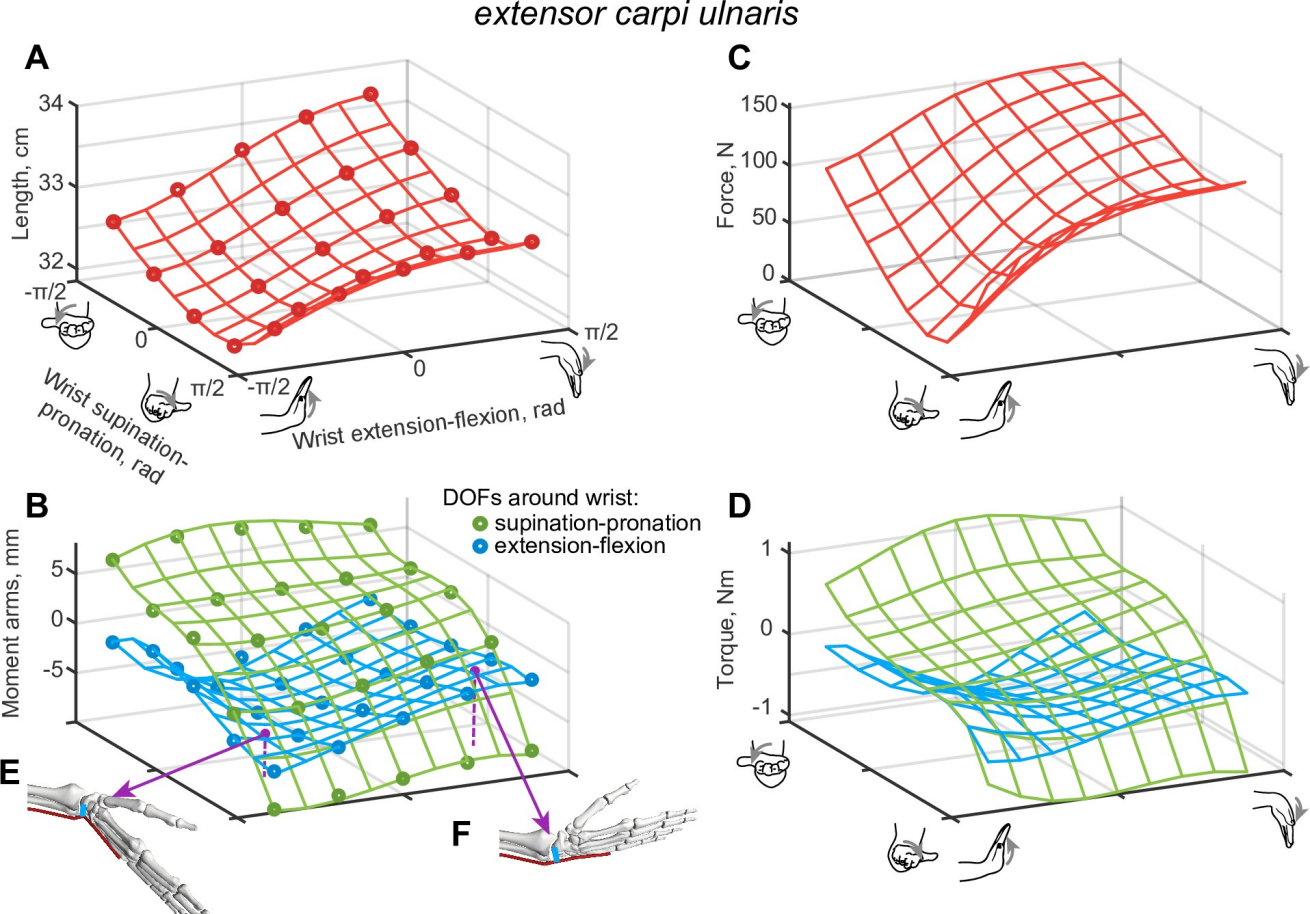
The example of kinematic approximation for *extensor carpi ulnaris*. **A**. The muscle path length is shown as a function of wrist e-f and s-p DOFs, with the continuous functions (plotted as wireframes) fitted into the data points from OpenSim model. **B**. The two corresponding moment arm relationships are shown for the same domain of postures. **C-D**. The maximal isometric muscle force and torque generation is shown for the same postures (see Eq 1–2 for details). **E**-**F**. Anatomical inserts showing muscle path (red) and moment arms for extension-flexion (blue) in two postures.

Moment arms and muscle lengths were approximated with a polynomial described by Eq 1.

$$f(x)=a+\sum_{p}^{\rho }\sum_{i_{1}\leq i_{2}\leq ..\leq i_{p}}^{d}K_{i_{1},i_{2},..,i_{p}}\prod_{j}^{p}x_{i_{j}},$$ (1)

where *a* is an intercept, *ρ* is the selected maximum of polynomial power, *d* is the number of DOFs, *x* = (*x*_1_,..,*x*_*d*_)^*T*^ is the state vector with values of angles at each DOF, *K* is the multidimensional matrix of polynomial term coefficients, sum and product coefficients (*p*, *i*, and *j*) iterate from 1. Indices *i*_*j*_∈[1;*d*] identify the coordinate $x_{i_{j}}$ that comprises the polynomial term $\prod_{j}^{p}x_{i_{j}}$, and the second sum combines all polynomial terms of power *d*. For example, *extensor carpi ulnaris* extension-flexion moment arm (with *ρ* = 4, *d* = 2) was approximated by Eq 2 (Fig 2B).

$$\mu_{1}(x)=a+K_{1}\cdot x_{1}+K_{2}\cdot x_{2}+K_{11}\cdot x_{1}^{2}+K_{12}\cdot x_{1}\cdot x_{2}+K_{22}\cdot x_{2}^{2}+K_{111}\cdot x_{1}^{3}+K_{112}\cdot x_{1}^{2}\cdot x_{2}+K_{122}\cdot x_{1}\cdot x_{2}^{2}+K_{222}\cdot x_{2}^{3}+K_{1112}\cdot x_{1}^{3}\cdot x_{2}+K_{1122}\cdot x_{1}^{2}\cdot x_{2}^{2}+K_{1222}\cdot x_{1}\cdot x_{2}^{3}+K_{2222}\cdot x_{2}^{4}.$$ (2)

where wrist extension-flexion moment arm *μ*_1_(*x*) in [mm] units was expressed as a function of the two corresponding DOF angles: wrist extension-flexion *x*_1_ and wrist supination-pronation *x*_2_ (in radians). Polynomial coefficient values for Eq 2: *a* = −5.43, *K*_1_ = 2.14, *K*_2_ = 1.09, *K*_11_ = 2.27, *K*_12_ = 1.23, *K*_22_ = 0.69, *K*_111_ = −1.23, *K*_112_ = −1.27, *K*_122_ = 0.41, *K*_222_ = 0.16, *K*_1112_ = 0.42, *K*_1122_ = −0.41, *K*_1222_ = −0.5, *K*_2222_ = −0.12.

The **polynomial structure** is then defined by the non-zero values of *K* and *a* parameters. Returning to the previous example, *extensor carpi ulnaris* moment arm was described by the following polynomial structure:

$$Polynomial\;structure(\mu_{1})=(a,K_{1},K_{2},K_{11},K_{12},K_{22},K_{111},K_{112},K_{122},K_{222},K_{1112},K_{1122},K_{1222},K_{2222});$$ (3)

Polynomial terms *K* use sorted indices to uniquely define them within polynomial structures (see Eq 3). For example, $x_{1}^{2}x_{2}$ is uniquely represented by *K*_*112*_, or *x*_1_*x*_1_*x*_2_. The sorting of indexes in *K* from low to high power forces other terms, e.g., *K*_*121*_ and *K*_*211*_, to collapse into the unique term *K*_*112*_. Examples of polynomial terms and corresponding structures are given in Table 1.

**Fig 3:**
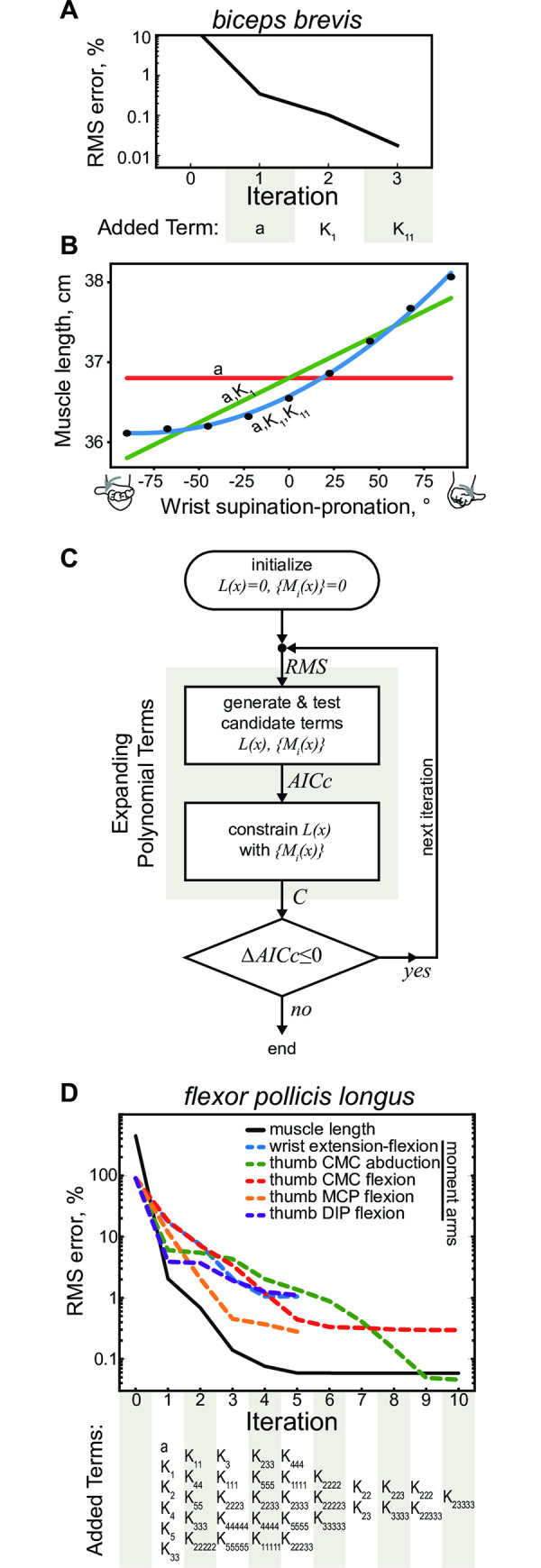
Optimal model generation algorithm. **A**. Example of generating a single polynomial structure to approximate the length of *biceps brevis*, which only crosses a single DOF (wrist s-p) in the model. **B**. Experimental *biceps brevis* length measurements (black circles) were sequentially fitted in three iterations (1: red; 2: green; 3: blue). **C**. The optimization flow schematic showing the flow of calculations using the amalgamated algorithm of model generation with physical constraint. RMS errors of model performance are computed at the onset of each new iteration and followed by the expansion of polynomial candidates. The process continues while there are improvements in AICc metric. **D**. Example of generating the system of polynomial functions describing *flexor pollicis longus*. The decrease in RMS errors for all DOFs actuated by this muscle were plotted for each iteration of the algorithm. The progression of terms added to minimize AICc in 6 polynomials is shown below the plot.

The example to clarify the sequential process of generating the approximation structure is illustrated in Fig 3. The errors in the approximation of *biceps brevis* in Fig 3A decrease with every additional term in the polynomial structures shown in Fig 3B. The general flow is further illustrated in Fig 3C with a complex example muscle *flexor pollicis longus*.

### Model physical constraints

Moment arms can be estimated as a partial differential of the muscle length in local coordinates [23,35]:

$$M_{i}(x)=\frac{\delta L(x)}{\delta x_{i}},$$ (4)

where *i* is the index of a DOF actuated by the muscle, *x*_*i*_ is the coordinate of *i*th DOF, *M*_*i*_(*x*) is the posture-dependent function of the moment arm around *i*th DOF, *L*(*x*) is the muscle length function. The kinematic variables of a given muscle are then captured by a single function *L*(*x*) and a set of functions {*M*_*i*_(*x*)} for muscles spanning multiple DOFs.

The following algorithm finds a new function *L*(*x*) and updates its set of moment arm functions {*M*_*i*_} in agreement with the relationship in Eq 4:

1. Calculate a set of intermediate muscle length polynomials ${\{\tilde{L}}_{i}(x)≔\int M_{i}dx_{i}\}$.
2. Combine the terms of *L*(*x*) and ${\{\tilde{L}}_{i}(x)\}:L(x)≔L(x)⋃(\underset{i}{⋃}{\{\tilde{L}}_{i}(x)\})$.
3. Differentiate analytically the polynomial *L*(*x*) (Eq 4) to update the complimentary set of moment arm functions, {*M*_*i*_(*x*)}.
4. Calculate *a* and *K* coefficients in *L*(*x*) and {*M*_*i*_(*x*)} using the original dataset.

For example, for an arbitrary muscle spanning two DOFs *x* = (*x*_1_, *x*_2_) with its length described by a function $L=2x_{1}x_{2}^{2}$, we have a polynomial term *x*_1_*x*_2_*x*_2_, which is denoted by the term *K*_122_. Similarly, the corresponding two moment arm functions $M_{1}=3x_{1}^{3}+2$ and *M*_2_ = 5*x*_1_*x*_2_ are described by the terms (*K*_111_, *a*) and (*K*_12_). The integrals of *M*_1_, *M*_2_ in step 1 are: ${\tilde{L}}_{1}=x_{1}^{4}+2x_{1}+const$ or structure (*a*, *K*_1_, *K*_1111_); ${\tilde{L}}_{2}=2.5x_{1}x_{2}^{2}+const$ or structure (*a*, *K*_122_). In step 2, the function *L*(*x*) adhering to Eq 4 is made by joining the terms of previous *L* with both ${\tilde{L}}_{1}$ and ${\tilde{L}}_{2}$, so that new $L=C_{0}+C_{1}x_{1}x_{2}^{2}+C_{2}x_{1}^{4}+C_{3}x_{1}$, where *C*_*i*_ are scalar coefficients in the structure (*a*, *K*_1_, *K*_122_, *K*_1111_). This step embeds the differential relationship between path length and its moment arms. In step 3, the moment arms are $M_{1}=C_{4}x_{2}^{2}+C_{5}x_{1}^{3}+C_{6}$ or structure (*a*, *K*_22_, *K*_111_) and *M*_2_ = *C*_7_*x*_1_*x*_2_ or structure (*K*_12_). We introduce this additional notation for constants to separate them from polynomial structures. We used a linear pseudoinverse on the original dataset to calculate the coefficients *C*_0−7_. These coefficients were used to evaluate the quality of fit (next section) and to analyze the nature of embedded information within the polynomials (see below, Kinematic Muscle Invariants).

### Model generation and validation

The geometries of muscle wrapping around joints vary greatly in their complexity and, consequently, their model representations. The simplest muscles can be approximated with a constant if their path is posture independent, and complex muscles may involve many polynomial terms. The search for the optimal model, as defined by the choice of functions and the criteria of optimality, requires the evaluation of each additional term from the domain of terms that grows exponentially with the number of actuated DOFs. Thus, muscles crossing 6 DOFs in our model were the most challenging. To solve this, we created an optimization algorithm similar to forward stepwise regression [36]. This method was adapted to include all possible polynomial terms and the constraint in Eq 4 in the process of expanding the polynomial structure with additional terms until the information tradeoff indicated overfitting. For this purpose, we used the corrected Akaike Information Criterion (AICc) for a finite sample size [37,38]:

$$AICc(f)=AIC(f)+\frac{2k(k+1)}{N-k-1}=2k-2\;\ln\left(L\right)+\frac{2k(k+1)}{N-k-1}$$ (5)

where *f* is an approximation function, *AIC* is the Akaike Information Criterion, *k* is the number of parameters in the model, *N* is the number of data points, and *L* is a maximum likelihood estimation of the polynomial representing this dataset. The peak value of *L* for the normally distributed estimated residuals is *ln*(*L*) = −0.5*N*(*ln*(2*πσ*^2^)+1) = −*N ln*(*σ*)+*const*, where *σ* is the root-mean-square (RMS) error. The model-independent constants are ignored in the substitution of *ln*(*L*) in Eq 5 because we use AICc values to compare multiple models (see further details on pp. 62–67 in [38]:

$$AICc(f)=2k+2N\;\ln\left(\sigma \right)+\frac{2k(k+1)}{N-k-1}$$ (6)

To remove potential differences between DOFs, we normalized the muscle length values to the range of motion and the moment arm values to their maximum across all physiological postures.

The analysis selected the terms of the polynomial structure for a muscle as follows (Fig 3C):

1. Initialize a variable (empty polynomial without terms) for the functions approximating muscle length *L*(*x*) and its set of moment arm functions, {*M*_*i*_(*x*)}.
2. Make a list of potential candidates for the expansion of each polynomial using all possible combinations from the fifth degree polynomial: *Ψ*(*L*); {*Ψ*(*M*_*i*_)}_*i*_.
3. Select optimal functions indicated by the smallest AICc values from the lists *Ψ*(•) and append them to the current approximation: $L(x)=\underset{f\in [\Psi (L);L]}{\mathrm{argmin}}AICc(f)$, $M_{i}(x)=\underset{f\in [\Psi (M_{i});M_{i}]}{\mathrm{argmin}}AICc(f)$.
4. Use the algorithm, described above (Model Physical Constraints), to impose the relationship of Eq 4.
5. Return to step 2: i) if further expansion is possible (*Ψ*(*L*) or *Ψ*(*M*_*i*_) are not empty), and ii) the change in AICc values is negative between iterations.

The progression of model assembly with this algorithm can be seen in Fig 3D showing the optimization of kinematic variables for *flexor pollicis longus* with the iterative expansion. The first evaluation of errors was performed relative to the zero model, where *L*(*x*) = 0; {*M*_*i*_(*x*)} = 0. The errors for the selected terms were evaluated in the following iteration step. In the first iteration, the muscle length was approximated by (*a*, *K*_1_, *K*_2_, *K*_4_, *K*_5_, *K*_33_), where some terms came from the selection of terms in step 3 and the rest from the integration in step 4. In the second iteration, the approximation was expanded using elements *K*_11_, *K*_44_, *K*_55_, *K*_333_, *K*_2222_, and the precision of muscle length fit decreased below 1%. In the fifth iteration, only thumb carpometacarpal (CMC) & metacarpophalangeal (MCP) moment arms required further optimization when other DOFs reached the minimum of AICc. In the tenth iteration, the evaluation of optimal parameter selection was finished with the high precision of 10^−3^ for the fit of muscle length across all physiological postures. Here, the worst moment arm fit of wrist extension-flexion (dashed blue line) was 1.05% in units normalized to the range of motion and the maximum magnitude of moment arm or 0.2 mm in absolute units.

The accuracy of a polynomial fit generally increases with the number of terms in the polynomial structure. For each iteration, the selection of potential candidates for expansion, Ψ(*P*(*x*)), contains polynomials with all terms of *P*(*x*) and one additional term from the possible additional terms in a polynomial of the same power. For example, let *P*(*x*) be a two-dimensional polynomial with structure (*a*, *K*_1_, *K*_11_), full 2-dimensional polynomial of power 2 has a structure (*a*, *K*_1_, *K*_2_, *K*_11_, *K*_12_, *K*_22_). Then the list of potential candidates is: *Ψ*(*P*(*x*)) = [(*a*, *K*_1_, *K*_2_, *K*_11_); (*a*, *K*_1_, *K*_11_, *K*_12_); (*a*, *K*_1_, *K*_11_, *K*_22_)]. The size of Ψ(*P*(*x*)) increases when higher power terms are required.

### Similarity index

Muscles with similar function may require similar approximation structures to capture their kinematics. To test this idea, we used a measure of similarity between polynomial structures. Consider polynomials *L*_*A*_ and *L*_*B*_ characterizing muscles A and B. Each polynomial can be described by a collection of shared or common terms (*P*_*C*_) and a collection of non-common terms (*P*_*NC*_), so that *L*_*A*_ = *P*_*C*_⋃*P*_*ANC*_ and *L*_*B*_ = *P*_*C*_⋃*P*_*BNC*_, where *P*_*ANC*_ are the terms present in *L*_*A*_ and not in *L*_*B*_ and *P*_*BNC*_ are the terms present in *L*_*B*_ and not in *L*_*A*_. Then, the similarity index (SI) is calculated as:

$$SI(A,B)=\frac{N_{C}}{N_{ANC}+N_{BNC}+N_{C}}\cdot 100\%$$ (7)

where *N*_*C*_, *N*_*ANC*_, *N*_*BNC*_ are the number of terms in *P*_*C*_, *P*_*ANC*_, *P*_*BNC*_, respectively. *SI* equals to 100% when two polynomials have completely identical structures (*K* terms), and to 0% when they are completely different.

### Kinematic muscle invariant

Additional details describing polynomial composition was captured using muscle representation in a Euclidean space formed by the basis of unique polynomial power terms (*K, Table 1*). Here, the obvious similarity due to mechanical actions around the same DOFs was removed (using *v*-axis index, described below and in Table 1) to test if the approximations contained additional functional relationships. Whether or not functional information is embedded in the pattern of polynomials could then be tested by examining the distance between muscles in this space. For the full polynomial of power *ρ* = 5 and maximum muscle dimensionality *d* = 6 these unique combinations are the following: [(1, 1, 1, 1, 1), (1, 1, 1, 1), (1, 1, 1, 2), (1, 1, 1), (1, 1, 2), (1, 1, 3), (1, 1), (1, 2, 2), (1, 2), (1, 3), (1, 4), (1), (2, 2), (2, 3), (2), (3), (4), (5)], where (1, 1, 1, 1, 1) is, e.g., *x*_1_*x*_2_*x*_3_*x*_4_*x*_5_ and (5) is $x_{i}^{5}$. The coefficients for these ordered 18 combinations defined the coordinates of a vector representing a given muscle-length polynomial. We converted all polynomials into unit vectors with the normalized sums of coefficients of the same terms from different DOFs, $\hat{v}={\left(v_{1},\ldots ,v_{n}\right)}^{T}/\|{\left(v_{1},\ldots ,v_{n}\right)}^{T}\|$. For example, for $L=C_{1}x_{1}x_{2}^{2}+C_{2}x_{1}^{2}x_{2}+C_{3}x_{1}^{3}+C_{4}x_{1}+C_{5}x_{2}+C_{6}$, the vector has nonzero elements [*v*_9_ = |*C*_1_|+|*C*_2_|; *v*_12_ = |*C*_4_|+|*C*_5_|; *v*_16_ = |*C*_3_|]. Structural difference of two polynomials can then be obtained as a distance between their vectors. We call vectors of each muscle in the basis of *v-*axes as *muscle invariants*. The structural difference between muscles is minimal when power composition of all terms and their absolute coefficients are similar in both polynomials even if they cross different DOFs, and large when their power compositions do not have the same terms.

### Musculoskeletal variables

To estimate the impact of approximation errors on the movement errors, we simulated musculoskeletal limb dynamics using Hill-type muscle models [24,39]. The contractile isometric force *F*(*L*) is generated by the contributions of active *F*_*a*_(*L*) and passive *F*_*P*_(*L*) forces that are expressed as functions of the muscle length *L*(*x*) (Eq 5 and Fig 2C) defined as the distance from origin to insertion on bones (Fig 2A).

$$\begin{array}{c} F(L)={u\cdot F}_{max}\cdot F_{a}(L)+F_{maxpass}\cdot F_{P}(L);\\ F_{a}(L)=2.5\cdot L_{norm}-1.25\cdot L_{norm}^{2};\\ F_{P}(L)=\left\{ \begin{array}{c} \frac{exp(2\cdot \frac{L-L_{pass}}{L_{max}-L_{min}})-1}{exp(1)-1},L>L_{pass}\\ 0,L\leq L_{pass} \end{array}.\right. \end{array}$$ (8)

The approximated function of muscle length *L*(*x*) is dependent on limb posture expressed as the state vector *x* = (*x*_1_,..,*x*_*d*_)^*T*^ with values corresponding to angles at each DOF. The length was normalized (*L*_*norm*_∈[0,1]) for the range within the full ROM using the shortest and longest values (*L*_*min*_, *L*_*max*_) for each muscle. The magnitude of passive and active force components were scaled by scalars *F*_*max*_ and *F*_*maxpass*_ = 0.1∙*F*_*max*_, respectively. The passive contribution increased exponentially when muscle length exceeded the passive tissue slack length *L*_*pass*_, which was set to 0.9∙*L*_*max*_. The muscle contraction level *u* was set to 1 in our analysis.

A muscle pulling on the bone segments produces rotational forces, or torques, at each DOF it actuates. The torque magnitude *τ* is defined by the moment arm *μ*, which is the shortest distance between the force vector along muscle path and the axis of rotation for each DOF (Fig 2B, 2E and 2F) expressed in scalar form in Eq 9.

τ(μ,L)=M·F(L), (9)

where *τ* = (*τ*_1_,..,*τ*_*d*_)^*T*^ and *M*(*x*) = (*μ*_1_(*x*),..,*μ*_*d*_(*x*))^*T*^ are arrays of torque and moment arm values for *d* DOFs actuated by a given muscle.

**Fig 4:**
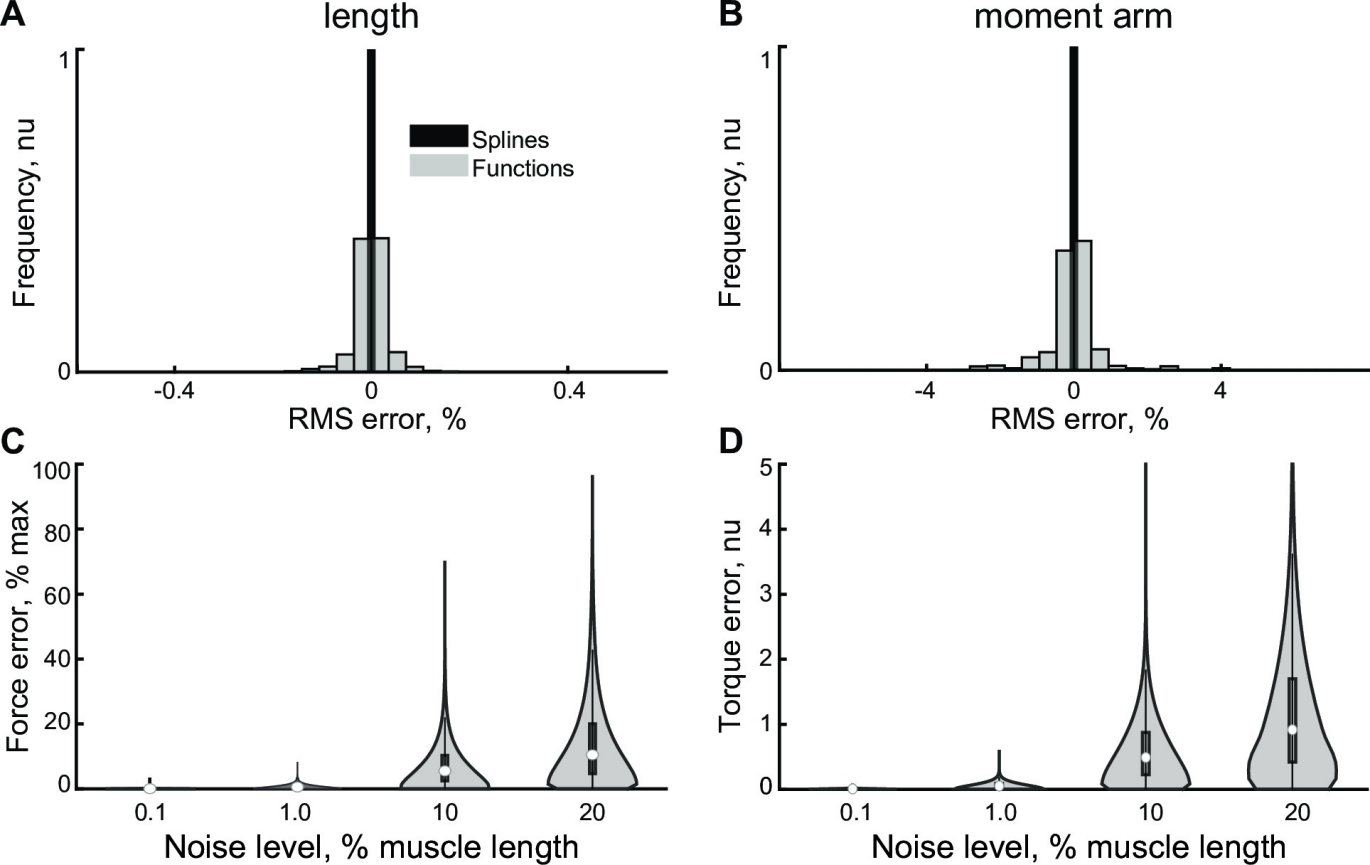
The distributions of normalized errors in the estimation of muscle lengths (**A**) and moment arms (**B**) are shown for two models (splines and polynomials). The histogram frequency was normalized to the total count of samples. **C**. Violin plots are showing the distribution of normalized error in maximum muscle force (**C**) and torque (**D**) in response to the noise muscle length profiles. Circles and boxes indicate medians and interquartile ranges, respectively.

The variation in muscle forces and moments resulting from the different levels of kinematic errors were estimated in maximal isometric contractions. For each of the possible 348,136 postures in the 8-point dataset, we calculated the kinetic reference dataset composed of muscle forces and torques computed with Eq 1 and 2. Next, we generated 10 sets of normally distributed muscle lengths and moment arms for each posture. The distribution was centered on the reference posture with randomly added deviation based on the standard deviation for the error perturbation. Using the magnitude differences between length and moment arm errors (identified in Fig 4A and 4B and Table 2), we assumed 10 times higher errors in muscle moment arms for a given level of error in muscle lengths. This allowed us to use a single parameter, the standard deviation of muscle length, as the perturbation magnitude. The generated muscle lengths were constrained within the physiological ROM. We selected four levels of errors: 1) based on the observed errors in the polynomial evaluations (about 0.1% of muscle length and 1% of moment arm); 2) 1%; 3) 10%, and 4) 20% of ROM. The latter two values are the expected kinematic errors without the rigorous data-driven profile matching, as identified in our model evaluation study [34]. The full perturbation dataset of random samples contained 3,481,360 muscle length and 19,450,000 moment arm values. The difference in muscle forces and moments were calculated based on the perturbation dataset and the previously computed kinetic reference dataset. The differences were normalized to the range of corresponding forces or moments in the kinetic reference dataset for a given muscle.

### Memory and time

The computer memory required for spline approximation was calculated as a size of MATLAB's ‘.mat’ files that contained single-precision spline parameters saved using '-v7.3' flag which enables compression. The computer memory required for polynomials was calculated as the size of executable ‘.mexw64’ files compiled with Visual Studio 2017 C++ with ‘/O2’ optimization. Evaluation time was obtained using MATLAB’s Profiler. Individual samples for mean and standard deviation of evaluation time were obtained per muscle’s dataset during estimation of fit quality. All computations were done on DELL Precision Workstation T5810 XL (Intel Xeon processor E5-2620 v3 2.4 GHz, 64 GB DDR4 RAM, SK Hynix SH920 512 GB SSD) running Windows 10.

### Statistics

The accuracy of polynomials was analyzed with standard statistical tools. The RMS error values were used to evaluate errors in the approximated values relative to the dataset used for fitting and the independent testing dataset (see above, Dataset). We detected outliers using a method similar to [29], by estimating maximum expected error (MEE, Eq 10) using Chebyshev’s theorem for the nonnormally distributed population and identifying the values outside of 99% of this distribution. This method caused the removal of less than 0.09% of values from both 9- and 8-point datasets.

$$MEE=\bar{\varepsilon }+\frac{1}{\sqrt{\alpha }}\sigma ,$$ (10)

where maximum expected error (*MEE*) was calculated as mean absolute error ($\bar{\varepsilon }$) and its standard deviation (*σ*) scaled by the selected significance level (*α*).

Linear regression was used to test the relationship between the complexity of functions represented by the number of actuated DOFs and the complexity of the approximating polynomials for each muscle. The muscle *ADPT* (S2 Table) was removed from this comparison using Tukey’s rule applied to the residuals of linear and exponential functions; only the residuals of ADPT exceeded median + 1.5 interquartile range (IQR).

The similarity of muscle invariants ($\hat{v}$) across multiple muscle groups was tested with dimensionality reduction analyses, i.e., principle component analysis (PCA) and hierarchical clustering. The Euclidean distance between vectors was first analyzed with the average linkage hierarchical clustering implemented in SciPy. Then, the dominant relationships in this distribution of *muscle invariants* were analyzed with PCA [Scikit-learn module in 40,41].

The representation of structural and functional information within the muscle length invariants was further tested by comparing the distributions of the distances between muscle pairs with similar structure or similar function to muscles with different structure or different function. These distributions were shown to be non-normal using D’Agostino’s K-squared test [42] that measures deviation from the normal skewness and kurtosis. We used one-tailed Mann-Whitney *U* test [43] to assess the two hypotheses that functional and structural similarities are represented in the colocalization of the *muscle invariants*. In general, this test was used to assess the likelihood of observing a smaller distance between the randomly selected pairs of *muscle invariants* with matching function or structure than the distance between the randomly selected pairs with shuffled function or structure. The smaller distances between the pairs in matched populations than the larger distances between the pairs from the shuffled populations were also tested with one-sided sign test [44]. The symmetrical distribution of samples around the mean is not assumed in the sign test; thus, it is a better choice for this problem then Wilcoxon signed-rank test. All tests were performed with a conservative value of α set at 0.01.

## Results

We developed a precise and efficient method to describe the MS kinematics of a human forearm and hand, extending previous work with approximation functions [26,29]. Here, we formalized the dynamic selection of terms in a best-fit polynomial function using a quantitative tracking of overfitting. Moreover, we used the differential relationship between muscle length and moment arms within the derivation algorithm to generate mutually consistent analytical models of these two variables. We tested if the composition of polynomials embedded information about muscle structure and/or function.

### Approximation of muscle lengths and moment arms

We subdivided values in the dataset (see above) into two groups to create and test the models. All best-fit models, splines and polynomials approximated moment arms with <5% error and muscle length with <0.4% error, as shown in Fig 4 and Table 2.

Although the approximation error with splines was the lowest, the implementation of splines required the highest number of parameters–eight orders of magnitude difference (compare cubic splines and constrained polynomials in Table 2). The large number of parameters in the cubic spline model exceeded the number of values in the dataset, which corresponded to impractical AICc values. We used AIC values instead to compare the relative quality of models; the constrained polynomial values were -6.7*10^6^ and -5.7*10^5^, as compared to the cubic spline values 2.2*10^9^ and 3.2*10^10^. This difference indicates the preference of AIC metric to the constrained polynomial model. The addition of model physical constraints (Eq 4) to the polynomial generation algorithm did not significantly change the precision of the polynomial model (p>0.9) with similar errors and AIC values (Table 2). The histograms of error distributions were superimposed (Fig 4). The length approximation errors (Fig 4A) were smaller than the moment arm errors (Fig 4B), as expected from Eq 4. In general, the differentiation process increased the error magnitudes.

A small portion of values in the datasets were marked as outliers and removed from further analyses: unconstrained polynomials had 0.08% muscle length outliers and 0.03% moment arm outliers; constrained polynomials had 0.08% and 0.03%, respectively. No spline errors were marked as outliers.

We simulated the propagation of errors from the computed kinematic MS parameters to the generated muscle force and torque values (Fig 4C and 4D). We found that force and torque error levels were negligible (mean error < 1%) for the lowest level of kinematic perturbation, but that further increases in kinematic errors may lead to kinetic errors that are an order of magnitude higher than the expected range (Table 3). The maximum torque errors for 10% and 20% perturbations were 6.4 and 12.5 times higher than the expected range of muscle torques.

**Table 3:** Estimated kinetic errors resulting from the expected kinematic errors.

Noise level	Force error, mean (IQR), % of range	Torque error, mean (IQR), % of range
0.1	0.0739 (0.0788)	0.0618 (0.065)
1.0	0.738 (0.787)	6.18 (6.49)
10	7.36 (7.8)	61.2 (64.6)
20	14.4 (15.3)	119 (128)

Both polynomial models were over 7000 times faster than the cubic spline (Table 4) and required 2.8*10^5^ times less memory. The search time for the constrained polynomials was 3.3 times faster than that for the unconstrained polynomials with the increase in performance gained when the selection of polynomial terms originated in the relationship between muscle length and moment arms.

**Table 4:** Time and memory requirements of approximations methods for kinematic variables.

Method	Evaluation, μs	Generation, min	Memory, KB
CS	7.8*10^4^± 0.7*10^4^	32	20.6*10^6^
UP	9.7±2.9	243	69
CP	9.9 ±2.0	74	73

### Structure of approximating polynomials

**Fig 5:**
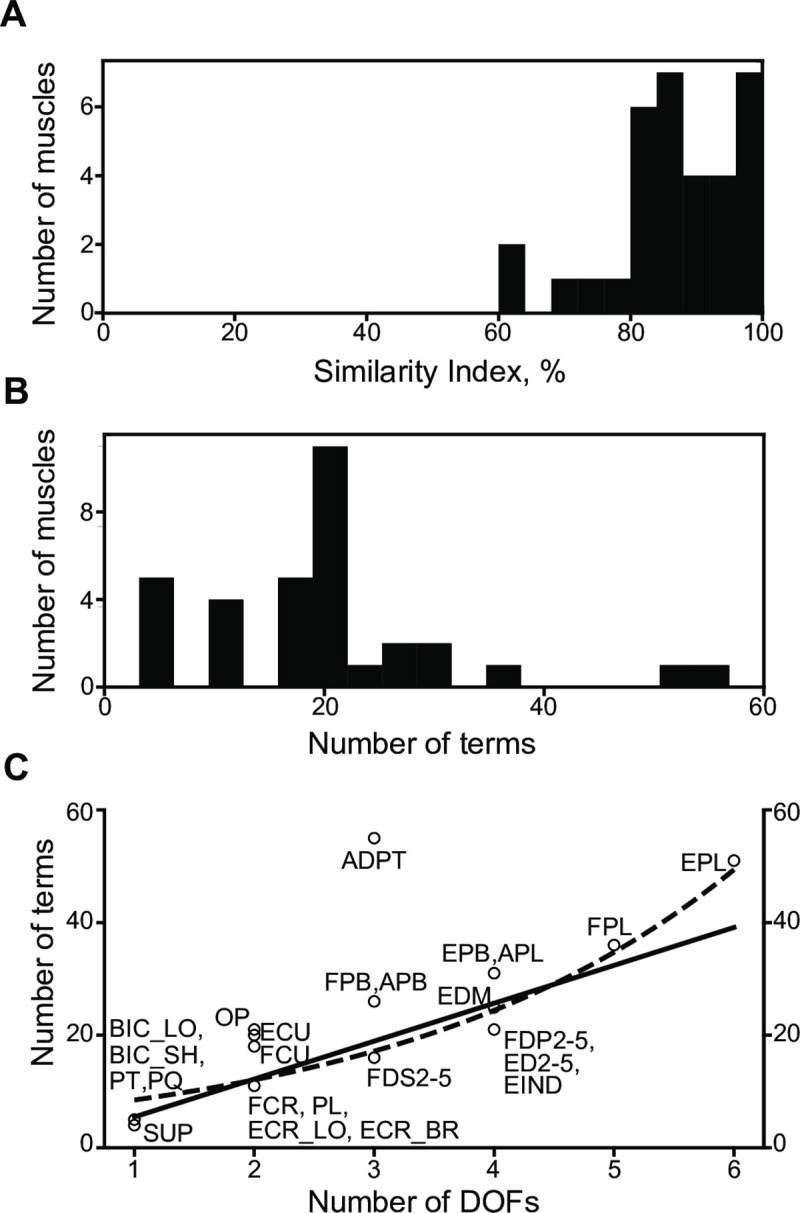
Complexity of muscle structures. **A.** Similarity index between functions approximating muscle lengths generated with and without the physical constraint imposed by Eq 6 in step 4 of the above algorithm. **B.** The distribution of polynomial complexity expressed as the number of terms. **C**. The relationship between the number of terms in the muscle length polynomial (circles) and the number of DOFs the muscle spans (solid line, *y* = 6.73*x*−0.12, *r* = 0.875, *p*<10^−8^; dashed exponent, *y* = 5.96∙*exp*(0.35*x*), *r* = 0.895, *p*<10^−8^). Several labels are attached to a single marker where data points superimpose.

Both the constrained and unconstrained polynomial models were similar in composition as determined by the high similarity between the two models (Fig 5A). Because the constrained muscle length function had higher polynomial power than its moment arm functions, we used *ρ* = 4 to generate Ψ(*M*_*i*_), and *ρ* = 5 to generate Ψ(*L*). The similarity index was high when both models contained the same polynomial terms, which was indicated by the predominance of high similarity indices for all muscles in Fig 5A. It took about 20 terms per muscle to achieve high accuracy (Fig 5B). The average similarity between muscles was 87.1%, and the biggest difference was observed in the three muscles *biceps brachii short head*, *flexor carpi radialis*, and *adductor pollicis transversus* with similarity indices at about 60%. This indicates that the compositions of constrained and unconstrained polynomial models were similar.

The increase in anatomical complexity indicated by the number of DOFs actuated by a muscle was predicted to correspond to the exponential increase in the number of terms required. This type of relationship was evident in the cubic spline model, where thumb muscles spanning up to 6 DOFs required the highest number of parameters. Conversely, it is remarkable that the relationship between the number of terms in the muscle length polynomial and the number of DOFs the muscle spans is instead linear (*r* = 0.87, Fig 5C solid line). The exponential approximation is similar to the linear result (*r* = 0.89, Fig 5C dashed line). Moreover, the model fractional complexity, measured as the ratio of terms selected to all possible terms available, decreased as the number of DOFs controlled by a muscle increased (S1 Fig, *r* = −0.88). Prior to calculating the regression, we removed an outlier, ADPT. Along with being a mathematical outlier, this muscle has unique anatomy. Similar to PT and PQ, ADPT is a short muscle, and unlike these muscles, ADPT crosses the thumb carpometacarpal joint, which is a geometrically complex biconcave-convex saddle joint that enables the characteristic dexterity of human prehension. The most complex muscles in our model were the thumb muscles (ADPT, FPB, APB, EPB, APL, FPL, EPL), and they appeared above the regression line (Fig 5C). Instead, the finger muscles (FDS2-5, FDP2-5, ED2-5, EDM, EIND) were below the regression line (Fig 5C), suggesting that these muscles have a lower relative complexity than the thumb muscles.

### Structure and function

**Fig 6:**
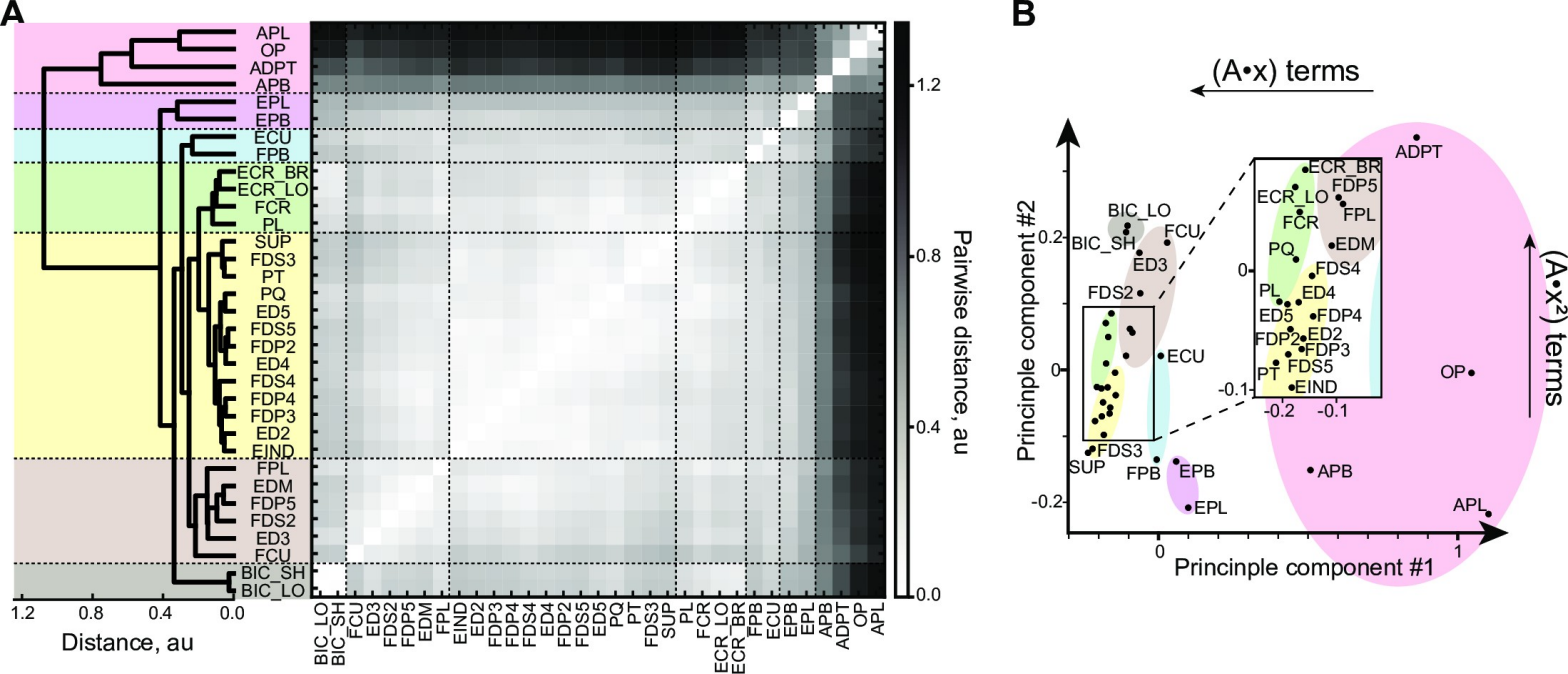
Kinematic muscle invariants. **A**. Average-linkage dendrogram computed from the heatmap of pairwise distances between muscle invariants. Horizontal dashed lines indicate subgroups described in text. **B**. The representation of muscle invariants in the space of their main two principle components. *Insert*: expanded view of a portion of the plot. The prevalence of linear and quadratic terms in polynomials is reflected within the principal directions (shown with arrows).

We hypothesized that the generated models capture structural and functional features of muscles and developed a measure of embedded muscle attributes, which we call *muscle invariants*. These muscle invariants represent each muscle in the space of polynomial term powers. The identification of physiological features with a combination of best-fit terms is expected from any sufficiently accurate model, even if this model is phenomenological (not mechanistic) in nature. To avoid trivial relationships where similarity could be simply determined by the index of DOF actuated by a pair of muscles, we removed DOF identity information and preserved only the power signature described by the power of variables within each term. This allowed us to focus on the dynamical properties common between muscles. The difference between muscles was captured as Euclidean distances between their vectors. To visualize the 18-dimensional space of all power terms (Table 1), the distance heatmap was calculated between all muscle pairs (Fig 6A), and the corresponding vectors were plotted in the axes of two main principle components computed with PCA (Fig 6B). The clustering algorithm generated the dendrogram based on these distances. A selection of distal thumb muscles (red: ADPT, APB, OP, APL) was visibly separated from about 6 other subgroups, with the closest subgroup formed by another subset of thumb muscles (purple: EPL and EPB). These groups were separated by the dashed line in the dendrogram of Fig 6A. The thumb muscles were followed (top to bottom) by: *extensor carpi radialis* and wrist flexors (green: ECR_LO, ECR_BR, FCR, PL), *flexor pollicis brevis* and *extensor carpi ulnaris* (blue: FPB and ECU), finger and wrist flexors and extensors, wrist rotators located in the forearm (yellow: FDP2-4, FDS3-5, ED2, ED4, ED5, EIND, PL, FCR, PQ, PT, SUP), the rest of digit muscles with *flexor carpi ulnaris* (brown: ED3, EDM, FDS2, FDP5, FCU, FPL), and *biceps brachii* (grey: BIC_SH, BIC_LO).

The differences between muscle invariants were largely captured by the first two principal components (86% of variance explained). Their largest coefficients were associated with linear (${\hat{v}}_{\left\{x\right\}}=-0.68$) and square (${\hat{v}}_{\left\{x^{2}\right\}}=0.84$) powers of polynomial terms. The linear relationship between joint angle and muscle length corresponds to a semi-circle muscle path around a joint. This simplistic behavior is characteristic for 1-DOF finger joints, muscles shown in the bottom-left corner and the insert of Fig 6B. Muscles in the bottom-right corner of Fig 6B, e.g., thumb muscles, used fewer linear terms than other muscles. Overall, the space of muscle invariants has a nonrandom and hierarchically structured pattern.

**Fig 7:**
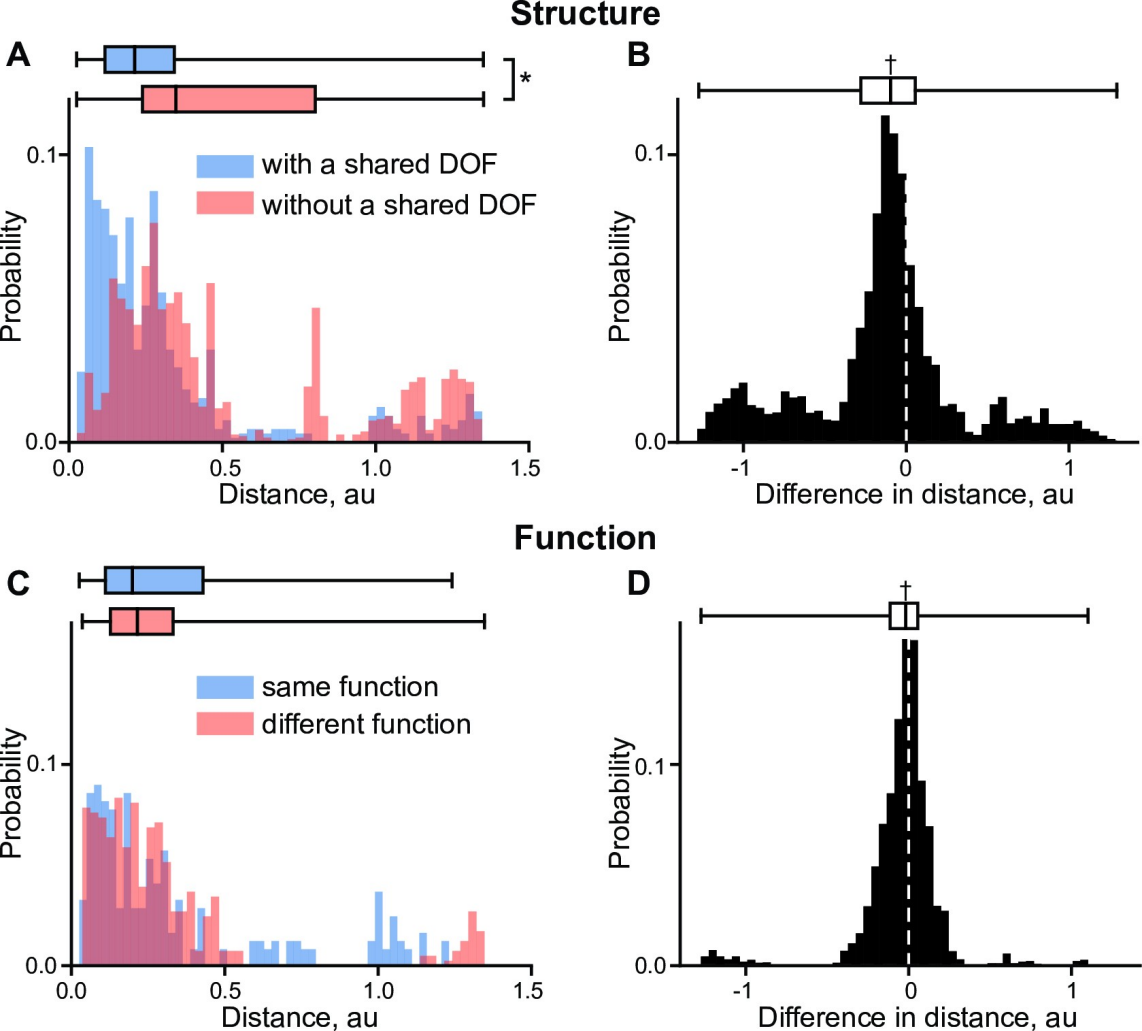
The structural and functional information embedded in muscle invariants. **A**. The probability distributions of observing the distance between the pairs of muscle invariants with (blue) and without (red) a shared DOF. **B**. The test of difference between the two groups. **C**. The probability distributions of the distance between the pairs with the shared structural information and with (blue) and without (red) shared functions. **D**. The test of difference between the two groups. Box plots indicate a median and 25^th^-75^th^ quantile region. The significant differences between the overlap of distributions tested with Mann-Whitney U test is marked with (*). The sign test significance is marked with (†).

We tested if muscle invariants contain information about their anatomical location by comparing Euclidian distances between the invariants with shared DOFs. Since there is a limited set of muscles that do not span the same joints, we tested the idea that those pairs of muscles that share a given DOF would be closer to each other than those that do not share that DOF. We assigned phalangeal DOFs (MCP, PIP, DIP) to be different to each other, but the same across fingers 2–5 because of their similarity and the lack of intrinsic hand muscles (e.g., lumbricals) in the model. This selection ensured local structural similarity in the group with a shared DOF (Fig 7A, blue) and local difference in the group without a shared DOF (Fig 7A, red), but it did not prevent the selection of muscle pairs in each group based on their structure relative to other DOFs. Fig 7A shows the probability of observing a given distance between a pair of muscles with a shared DOF and without a shared DOF based on 653 and 1862 pairs, respectively. The selection of muscles into these groups was executed sequentially by examining all muscles for each DOF in the model. The pairs of muscles were selected once at each DOF. The difference distribution between the two distributions in Fig 7A, shown in Fig 7B, was computed by examining the difference between each pair with a shared DOF and subtracting from it each pair that had one of the two muscles in the group without a shared DOF. This produced the population consisting of 10,373 values, which was then compared to zero by a single test. The median difference was significantly different from zero (-0.10, sign test *p*<10^−8^), with 68% of values in the difference population being less than zero. Both groups were not normally distributed (D’Agostino’s K-squared test of normality, *p*<10^−8^) and similar anatomical pairs were closer to each other, which was evident from the non-equal distribution of the two groups (Mann-Whitney U test: *U* = 3.7∙10^5^, *p*<10^−8^). We found that the muscle invariants capture the structural information related to the identity of their actuated DOFs.

We tested if the muscle invariants contain functional information beyond that explained by the anatomical similarities. For this purpose, we defined seven functional categories based on their primary mechanical function: wrist supinators (BIC_LO, BIC_SH, SUP), pronators (PT, PQ), extensors (ECR_LO, ECR_BR, ECU), flexors (FCR, FCU, PL), finger flexors (FDS2-5, FDP2-5), extensors (ED2-5, EDM, EIND), and thumb muscles (APL, OP, APB, EPL, EPB, FPB, FPL, ADPT). We tested the idea that two muscles from the same category are closer together than those from different categories even when all these muscles actuate the same DOF. For this reason, we selected all pairs of muscles with (245 pairs) and without (816 pairs) a shared function selected from the seven categories and computed the distance between these pairs, shown in Fig 7C. The pairs of muscles were selected once at each combination of DOF and functional cluster. Next, we computed the distance between the two groups based on the combinations of all these pairs (1748 samples), shown in Fig 7D. The distributions in Fig 7C were also not normal (*p*<10^−8^). While distributions of the two groups were overlapping (*p* = 0.59), the median difference between them was significantly different from zero (-0.01, sign test *p*<10^−4^). Although the effect size is small, this supports the hypothesis that DOF-independent functional differences are captured by the polynomial structure.

## Discussion

We approximated MS kinematics of the human forearm and hand with a new type of autogenerating model that embeds biomechanical constraints between muscle parameters. The model reached optimal performance with polynomial simulations showing both high precision and computational efficiency. While the model was developed as a descriptive tool, the fine details captured within the muscle-posture relationships include the differential connection between moment arms and muscle lengths and reflect the high-level mechanistic properties of forearm and hand muscle function. The composition of terms in these models was objectively determined by the embedded information and demonstrated the patterns associated with anatomy and function, which is an indication of high approximation accuracy in our phenomenological model. The mechanical specification of muscles for the control of different hand DOFs and different functions has not been previously demonstrated.

All models are simplifications or approximations of reality, but some approximations are useful [45]. The complex geometric interactions—sliding and wrapping—between muscles and other mechanical body structures pose a considerable computational challenge for real-time applications [46]. Typically, the engineering trade-off between complexity, performance, and accuracy pushed development towards simplified biomechanical limb models that assumed constant moment arm and posture relationships [18] or models that used the approximations of muscle kinematics to ease computational demands [30]. Here, we report a new method of capturing the kinematic MS transformations within the biomechanical model of the forearm and hand that further improves the process of developing accurate MS models for real-time applications. Moreover, the objective system identification of model structure captured structural and functional features of MS organization indicating that this phenomenological model captures accurately mechanistic details without their explicit use in the generation process.

### Autogenerating models

Interest in MS approximations has been steadily increasing with the development of computational tools for human motion analysis, e.g., OpenSim [25]. The accuracy of these approximations has been demonstrated with B-spline models [29,30] and computational efficiency has been achieved with polynomial models [26,28]. The optimal polynomials derived here have the benefits of being both accurate and computationally efficient.

The manual subjective selection of polynomial terms for each muscle is usually based on the number of DOFs the muscle crosses, the quality of simulation, and the numerical cost of evaluating functions. In contrast, our optimization algorithm chooses the polynomial terms objectively based on the information criterion to reflect dependencies within the data. The information criterion is a type of cost function that allows comparison between different polynomial models and prevents overfitting with an excessive number of terms. The latter is possible when using the subjective desired precision of fit, as in [28]. We selected the polynomial form of approximations because the parameters can be efficiently determined with linear pseudoinverse and to extend the previous studies with similar methodology. Similar to [26] the number of terms in the optimized polynomial grows with the number of muscle’s DOFs, but the term composition varies to reflect the diverse anatomy and function.

We found multiple levels of structure embedded in the power composition of polynomial terms. A linear relationship between muscle length and joint angle is characteristic for 1-DOF finger joints. The near-linear relationship between moment arm profile and joint angle we showed in thumb muscles has been commonly observed in other studies [26,47]. The physiological function of this relationship could be associated with compensation for the muscle force-length relationship at the edges of the range of motion. The diverse function and behavior of thumb muscles found during movement [48] is mirrored in our results by their separation from other muscles and high variability between each other.

Similar to the previous analysis, thumb muscles are clearly separated from other finger muscles. Previously we have examined the grouping of muscles based on their length-posture relationships where the similarity between muscles was determined by common muscle length shortening and lengthening in response to postural changes [see Fig 7 in 32]. The previous analysis used the negative sign of regressions in the relationship between muscle length and posture to separate antagonistic muscles. Still, we found differences in the composition of polynomials that were described by antagonistic muscle relationships. These differences (Fig 7C and 7D) were significant even when DOF identity, a confounding variable, was matched. The result indicated a functional difference between the muscle invariants even when the differences accounted for by muscle location were removed; albeit, this difference was small. The small effect size of the functional difference is possibly related to the subjective definition of the muscle function reflecting only the primary single joint actions. It is possible that this result may change if other types of approximations are used. Overall, this supports the idea that the commonly observed during movement muscle synergies can be at least in part explained by the specialization of structure and function embedded in the musculotendon paths.

### Real-time high-dimensional musculoskeletal computations

The optimal polynomials efficiently compute highly complex MS kinematics for real-time applications. The polynomials describing 33 musculotendon actuators each crossing up to 6 DOFs can be evaluated within 10 μs, requiring less than 75 KB of RAM. To contrast, the previous state-of-the-art performance for a lower-limb model with 13 musculotendon actuators, each crossing up to 3 DOF was shown to be less than 2.5 ms [30]. Our more than hundred-fold time efficiency improvement on the method was also accompanied by a similar improvement in required memory (about 10MB worth of coefficients in [30] based on [29]). The improvements are largely due to the exponential rise in the required computational resources with the dimensionality increase of the spline model, as previously shown [29] and by our implementation. This *dimensionality curse* may prevent the application of splines in complex models recently developed for offline analyses [49–51]. Our optimal polynomial approach shows linear scaling of the model (Fig 5C) allowing these models to be used in real-time applications.

The described optimization algorithm is structurally similar to stepwise regression [36], but has several important differences. First, it automatically constructs and explores all possible polynomial combinations of the input variables within reasonable power limitations. Second, our algorithm uses AIC [37,52] instead of F-statistic as the objective measure of improvements. The additional term in AIC takes into account the trade-off between the quality of fit and the increased model complexity. This is a novel use of information measures (Akaike, Bayesian and other) that have been previously used mostly as a stopping criterion [53]. An information criterion allows flexibility when choosing the tradeoff between quality of fit and the measure of model complexity. For example, using the number of processor commands instead of the number of variables for each term is useful for the development of extremely high-performing routines or for computationally costly devices, like portable chips or graphics processing units. Third, our approximation algorithm embeds the differential relationship between muscle length and its moment arms in the search for the best polynomial coefficients. This novel approach of using the formulation of structural constraints within the algorithm decreased model assembly time. These approximations are ready to be used on a portable device that requires a real-time simulation of MS variables, e.g., a biomimetic prosthesis or a medical assessment device.

### Limitations

We chose to implement the approximation algorithm with the use of polynomial sequences as the most accurate representation of the MS relationships. The alternative implementations could use sequences of trigonometric or exponential terms. For example, any data with periodic relationships would be efficiently represented by trigonometric functions, and any data with sigmoidal transitions or limits of range could be represented by exponential functions. However, the relationships between moment arms and posture for wrist and hand muscles are smooth because of viscoelastic soft tissue properties. In this case, we can rely on the theoretical conclusion from Taylor’s theorem stating that any smooth function can be described with a polynomial approximation. Then the only potential failure of our method would be a discontinuity in the muscle paths. We have indeed observed sharp transitions in simulated data; however, these observations were associated with geometric model failures. Typically, a muscle path slipped off a wrapping surface. These behaviors were detected and corrected prior to the approximation [33]. Thus, our model is appropriate for the physical system it represents.

Another potential limitation could be associated with the sequential selection of optimal polynomial terms. The autogenerating polynomials were iteratively created with the selection of a single term per equation at a time. This enabled fast optimization of the full system of equations describing moment arms and muscle lengths. It is theoretically possible that the selection of multiple terms at a time can be more optimal than their iterative sequential selection. This would be indicated by the premature termination of the optimization routine even when a more optimal solution is available for multiple terms selected in the same iteration. We tested this eventuality by repeating the model generation with an algorithm capable of adding one or two terms per iteration per equation. This method produced the same solutions for our dataset, but the evaluation time increased by an order of magnitude as compared to the standard method.

The musculoskeletal evaluation using the Hill-type muscle model is subject to the kinematic errors within experimental or simulation measurements of muscle length and its moment arms. Our perturbation analysis of these computations revealed that the predicted torques became substantially inaccurate (> 60%) for errors exceeding 10% of the physiological ROM. While the errors in muscle length can be bound by the limited range of muscle excursion along the path between its origin and insertion, the errors in moment arms are not constrained. In addition, the differential relationship between the muscle lengths and moment arms (Eq 4) exacerbates the problem if the moment arm relationships are derived from the typical measurements of muscle length profile for a range of posture. These results indicate that the fitting errors in this study (less than 1% in muscle length) generated tolerable errors in the simulations with dynamics.

The current method is limited to the description of forearm muscles in a generic representation of the human hand. Future analysis of validated models that span the shoulder will improve our understanding of muscle specialization. We expect to see complex functional groups with the possibility of observing the structure different from that of any of the hand functional groups because of the unique biomechanics of the shoulder joint [54,55]. These functional groups can be then further refined by their evaluation on models with subject-specific segment scaling and morphometric differences [56]. It will be also illuminating to compare the muscle organization of the upper limb to that of the lower limb, considering their proposed coevolution [57], covariability in developmental modules [58], and high observed topological similarity [59] in humans. However, accurate and valid lower-limb models are still under development. Overall, the examination of polynomial structure yields a new characterization method; yet, the encouraging implications of anatomical and functional representations within the polynomials still require further investigation.

## Conclusions

We approximated the kinematic variables for human hand and forearm muscles with both high precision (<5% error across 18 DOFs) and efficiency (<75 KB, <10 μs). Adding the differential relationship between moment arms and muscle lengths improved accuracy and the speed of model generation. The approach overcomes *the curse of dimensionality* and scales linearly with increased complexity for large MS models. The structural content of optimal polynomials reflects muscle anatomy and, to a smaller extent, function. This novel description can be further applied in neuromechanics and its applications.

## Supporting information

S1 Text (DOCX)

S1 Table The list of simulated DOFs. Each label describes both a DOF and the direction of axis using the following structure: <LIMB>_<JOINT>_<MIN>_<MAX>, where LIMB corresponds to the limb where the joint is located, i.e. ‘ra’ stands for ‘right arm’, JOINT is the joint of this DOF, e.g., ‘wr’ is ‘wrist’. Digit joints have their identifying number: 1 thumb; 2 index; 3 middle; 4 ring; and, 5 pinky. The last two suffixes MIN and MAX indicate the anatomical direction of axis, e.g., ‘ra_wr_s_p’ indicates the range of the wrist pronation-supination DOF (-1.5708 rad for the supinated posture and the maximum 1.5708 rad for the pronated posture).
(DOCX)

S2 Table The list of simulated musculotendon actuators. Brief labels used in figures are shown with their anatomical names and the corresponding information about the number and identity of actuated DOFs, as described in S1 Table.
(DOCX)

S1 Fig Complexity of muscle structures. A. The distribution of relative polynomial complexity expressed as the portion of parameter space used. B. The relationship between the relative complexity of the muscle length polynomial (circles) and the number of DOFs the muscle spans (line, *y* = 100−19.4*x*, *r* = −0.879, *p*<10^−8^). Relative complexity of a polynomial was estimated as a fraction of the parameter space that the polynomial occupies. For example, if the number of terms in the 2-dimensional polynomial is 3, and the size of the parameter space of 2-dimensional polynomial of power 2 is 6, and the relative complexity is 3/6 = 50%.
(DOCX)

S2 Fig Similarity of muscle structures using Similarity Index. Average-linkage dendrogram computed from the heatmap of pairwise Similarity Index. The distance between clusters was calculated as an average distance between elements of two clusters.
(DOCX)
