# Supplementary material for: Approximating complex musculoskeletal biomechanics using multidimensional autogenerating polynomials
Source: PLoS Comput Biol. 2020 Dec 16;16(12):e1008350. doi: 10.1371/journal.pcbi.1008350 (PMC7773415; doi:10.1371/journal.pcbi.1008350)
Supplement: S1 Text — (DOCX) [file pcbi.1008350.s001.docx]

### S1 Text

The validity of selecting the sampling rate of the relationship between posture and muscle parameters was tested by comparing the quality of approximation with three different rates, i.e., the training datasets were sampled at 3, 5, and 9 values per degree of freedom (DOF). The corresponding three testing datasets with data points residing between the training data points were used for validation. The overall fitting errors were not significantly different between 5- and 9-point datasets. However, infrequent failures in the 5-point model were effectively resolved with the 9-point model. It is likely that further increases in the sampling rate are not likely to increase model performance and may lead to the overfitting by exceeding the quality of the musculoskeletal representation in OpenSim. Since the 5-point model had a very similar performance to the 9-point model, it can be effectively used as an intermediate fast approximation for iterative adjustments needed to validate muscle geometry against experimental data [as in 1]. Overall, the 9-point model was deemed to be optimal.

1. Boots MT, Sobinov A, Gritsenko V, Mansoori BK, Fisher LE, Collinger JL, et al. Scaling of musculoskeletal morphometry for human upper-limb models. Society for Neuroscience Abstracts. Washington, D.C.; 2017. p. 642.03.
