# Supplementary material for: Approximating complex musculoskeletal biomechanics using multidimensional autogenerating polynomials
Source: PLoS Comput Biol. 2020 Dec 16;16(12):e1008350. doi: 10.1371/journal.pcbi.1008350 (PMC7773415; doi:10.1371/journal.pcbi.1008350)
Supplement: S1 Table — Each label describes both a DOF and the direction of axis using the following structure: ___, where LIMB corresponds to the limb where the joint is located, i.e. ‘ra’ stands for ‘right arm’, JOINT is the joint of this DOF, e.g., ‘wr’ is ‘wrist’. Digit joints have their identifying number: 1 thumb; 2 index; 3 middle; 4 ring; and, 5 pinky. The last two suffixes MIN and MAX indicate the anatomical direction of axis, e.g., ‘ra_wr_s_p’ indicates the range of the wrist pronation-supination DOF (-1.5708 rad for the supinated posture and the maximum 1.5708 rad for the pronated posture). (DOCX) [file pcbi.1008350.s002.docx]

### S2 Table

The list of simulated DOFs. Each label describes both a DOF and the direction of axis using the following structure: <LIMB>_<JOINT>_<MIN>_<MAX>, where LIMB corresponds to the limb where the joint is located, i.e. ‘ra’ stands for ‘right arm’, JOINT is the joint of this DOF, e.g., ‘wr’ is ‘wrist’. Digit joints have their identifying number: 1 thumb; 2 index; 3 middle; 4 ring; and, 5 pinky. The last two suffixes MIN and MAX indicate the anatomical direction of axis,

e.g., ‘ra_wr_s_p’ indicates the range of the wrist pronation-supination DOF (-1.5708 rad for the supinated posture and the maximum 1.5708 rad for the pronated posture).

| DOF ID | Label | Range  [MIN, MAX], rad | Description of action |
| --- | --- | --- | --- |
| 1 | ra_wr_s_p | -1.5708, 1.5708 | wrist pronation/supination motion |
| 2 | ra_wr_e_f | -1.2217, 1.2217 | wrist flexion/extension motion |
| 3 | ra_cmc1_f_e | 0, 0.8727 | thumb proximal flexion/extension motion |
| 4 | ra_cmc1_ad_ab | 0, 0.8727 | thumb proximal abduction/adduction motion |
| 5 | ra_mcp1_f_e | -0.7854, 0 | thumb central flexion/extension motion |
| 6 | ra_ip1_f_e | -1.5708, 0 | thumb distal flexion/extension motion |
| 7 | ra_mcp2_e_f | 0, 1.5708 | index proximal flexion/extension motion |
| 8 | ra_pip2_e_f | 0, 2.0944 | index central flexion/extension motion |
| 9 | ra_dip2_e_f | 0, 1.5708 | index distal flexion/extension motion |
| 10 | ra_mcp3_e_f | 0, 1.5708 | middle proximal flexion/extension motion |
| 11 | ra_pip3_e_f | 0, 2.0944 | middle central flexion/extension motion |
| 12 | ra_dip3_e_f | 0, 1.5708 | middle distal flexion/extension motion |
| 13 | ra_mcp4_e_f | 0, 1.5708 | ring proximal flexion/extension motion |
| 14 | ra_pip4_e_f | 0, 2.0944 | ring central flexion/extension motion |
| 15 | ra_dip4_e_f | 0, 1.5708 | ring distal flexion/extension motion |
| 16 | ra_mcp5_e_f | 0, 1.5708 | pinky proximal flexion/extension motion |
| 17 | ra_pip5_e_f | 0, 2.0944 | pinky central flexion/extension motion |
| 18 | ra_dip5_e_f | 0, 1.5708 | pinky distal flexion/extension motion |

The list of simulated DOFs. The last two suffixes separated by underscores indicate the direction of the motion, e.g., “_*s_p”* indicates the motion from supinated to pronated posture.
