# Supplementary material for: Approximating complex musculoskeletal biomechanics using multidimensional autogenerating polynomials
Source: PLoS Comput Biol. 2020 Dec 16;16(12):e1008350. doi: 10.1371/journal.pcbi.1008350 (PMC7773415; doi:10.1371/journal.pcbi.1008350)
Supplement: S2 Table — Brief labels used in figures are shown with their anatomical names and the corresponding information about the number and identity of actuated DOFs, as described in S1 Table. (DOCX) [file pcbi.1008350.s003.docx]

### S3 Table

The list of simulated musculotendon actuators. Brief labels used in figures are shown with their anatomical names and the corresponding information about number and identity of actuated DOFs, as described in S1 Table.

| Muscle ID | Label | Musculotendon actuator | Number of DOFs | DOF IDs |
| --- | --- | --- | --- | --- |
| 1 | BIC_LO | *Biceps brachii long head* | 1 | 1 |
| 2 | BIC_SH | *Biceps brachii short head* | 1 | 1 |
| 3 | SUP | *Supinator* | 1 | 1 |
| 4 | PT | *Pronator teres* | 1 | 1 |
| 5 | PQ | *Pronator quadratus* | 1 | 1 |
| 6 | ECR_LO | *Extensor carpi radialis longus* | 2 | 1 2 |
| 7 | ECR_BR | *Extensor carpi radialis brevis* | 2 | 1 2 |
| 8 | ECU | *Extensor carpi ulnaris* | 2 | 1 2 |
| 9 | FCR | *Flexor carpi radialis* | 2 | 1 2 |
| 10 | FCU | *Flexor carpi ulnaris* | 2 | 1 2 |
| 11 | PL | *Palmaris longus* | 2 | 1 2 |
| 12 | FDS5 | *Flexor digitorum superficialis (pinky finger)* | 3 | 2 16 17 |
| 13 | FDS4 | *Flexor digitorum superficialis (ring finger)* | 3 | 2 13 14 |
| 14 | FDS3 | *Flexor digitorum superficialis (middle finger)* | 3 | 2 10 11 |
| 15 | FDS2 | *Flexor digitorum superficialis (index finger)* | 3 | 2 7 8 |
| 16 | FDP5 | *Flexor digitorum profundus (pinky finger)* | 4 | 2 16 17 18 |
| 17 | FDP4 | *Flexor digitorum profundus (ring finger)* | 4 | 2 13 14 15 |
| 18 | FDP3 | *Flexor digitorum profundus (middle finger)* | 4 | 2 10 11 12 |
| 19 | FDP2 | *Flexor digitorum profundus (index finger)* | 4 | 2 7 8 9 |
| 20 | EDM | *Extensor digiti minimi* | 4 | 2 16 17 18 |
| 21 | ED5 | *Extensor digitorum (pinky finger)* | 4 | 2 16 17 18 |
| 22 | ED4 | *Extensor digitorum (ring finger)* | 4 | 2 13 14 15 |
| 23 | ED3 | *Extensor digitorum (middle finger)* | 4 | 2 10 11 12 |
| 24 | ED2 | *Extensor digitorum (index finger)* | 4 | 2 7 8 9 |
| 25 | EIND | *Extensor indicis* | 4 | 2 7 8 9 |
| 26 | EPL | *Extensor pollicis longus* | 5 | 1 2 4 3 5 6 |
| 27 | EPB | *Extensor pollicis brevis* | 4 | 2 4 3 5 |
| 28 | FPB | *Flexor pollicis brevis* | 3 | 4 3 5 |
| 29 | FPL | *Flexor pollicis longus* | 5 | 2 4 3 5 6 |
| 30 | APL | *Abductor pollicis longus* | 4 | 1 2 4 3 |
| 31 | OP | *Opponens pollicis* | 2 | 4 3 |
| 32 | APB | *Abductor pollicis brevis* | 3 | 4 3 5 |
| 33 | ADPT | *Adductor pollicis transversus* | 3 | 4 3 5 |
