# Supplementary material for: Approximating complex musculoskeletal biomechanics using multidimensional autogenerating polynomials
Source: PLoS Comput Biol. 2020 Dec 16;16(12):e1008350. doi: 10.1371/journal.pcbi.1008350 (PMC7773415; doi:10.1371/journal.pcbi.1008350)
Supplement: S1 Fig — A. The distribution of relative polynomial complexity expressed as the portion of parameter space used. B. The relationship between the relative complexity of the muscle length polynomial (circles) and the number of DOFs the muscle spans (line, y = 100−19.4x, r = −0.879, p<10−8). Relative complexity of a polynomial was estimated as a fraction of the parameter space that the polynomial occupies. For example, if the number of terms in the 2-dimensional polynomial is 3, and the size of the parameter space of 2-dimensional polynomial of power 2 is 6, and the relative complexity is 3/6 = 50%. (DOCX) [file pcbi.1008350.s004.docx]

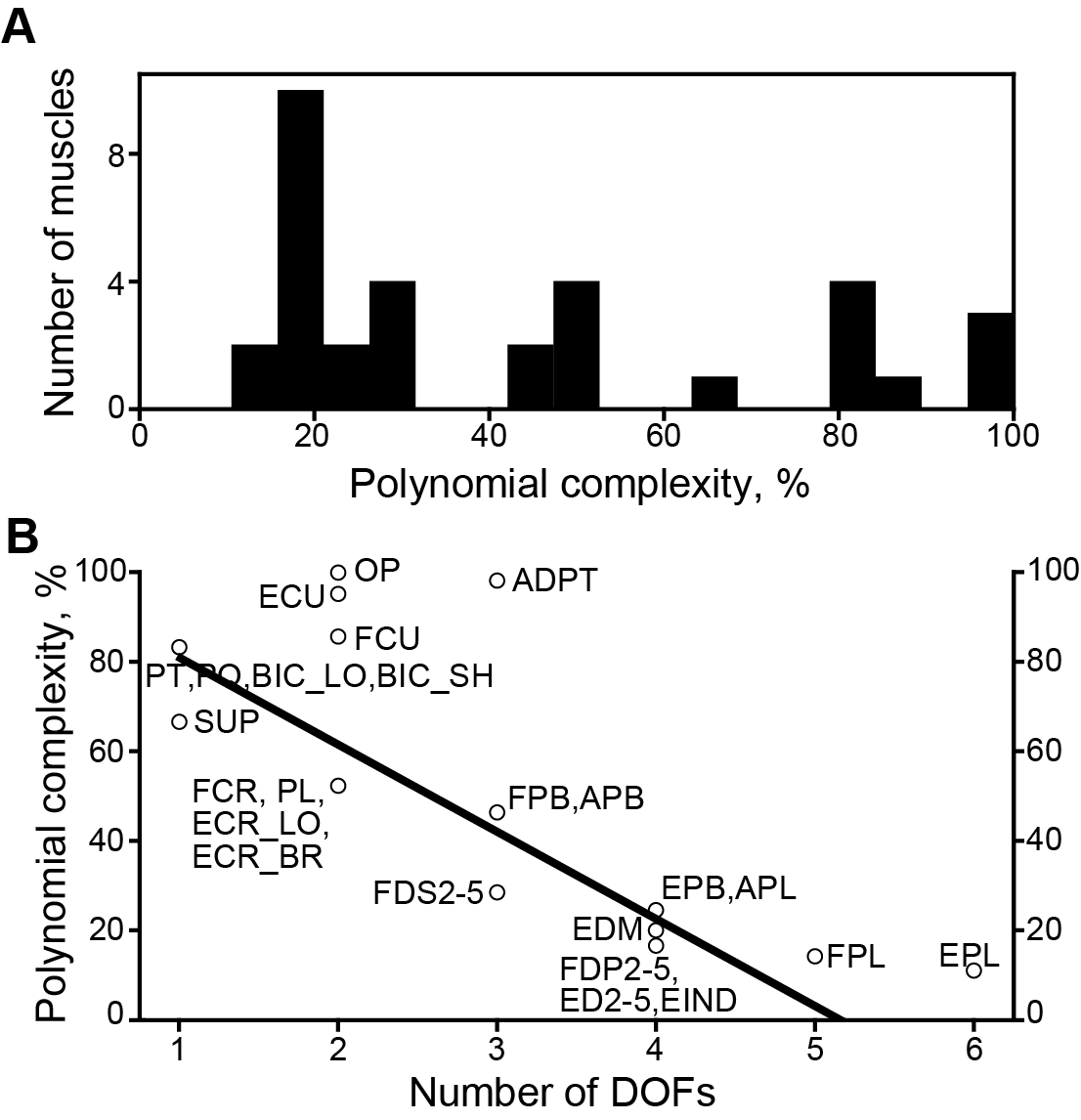


**S4 Figure**. Complexity of muscle structures. A. The distribution of relative polynomial complexity expressed as the portion of parameter space used. B. The relationship between the relative complexity of the muscle length polynomial (circles) and the number of DOFs the muscle spans (line, $y=100-19.4x$, $r=-0.879$, $p<{10}^{-8}$). Relative complexity of a polynomial was estimated as a fraction of the parameter space that the polynomial occupies. For example, if the number of terms in the 2-dimensional polynomial is 3, and the size of the parameter space of 2-dimensional polynomial of power 2 is 6, and the relative complexity is 3/6=50%.
