# Supplementary material for: Approximating complex musculoskeletal biomechanics using multidimensional autogenerating polynomials
Source: PLoS Comput Biol. 2020 Dec 16;16(12):e1008350. doi: 10.1371/journal.pcbi.1008350 (PMC7773415; doi:10.1371/journal.pcbi.1008350)
Supplement: S2 Fig — Average-linkage dendrogram computed from the heatmap of pairwise Similarity Index. The distance between clusters was calculated as an average distance between elements of two clusters. (DOCX) [file pcbi.1008350.s005.docx]

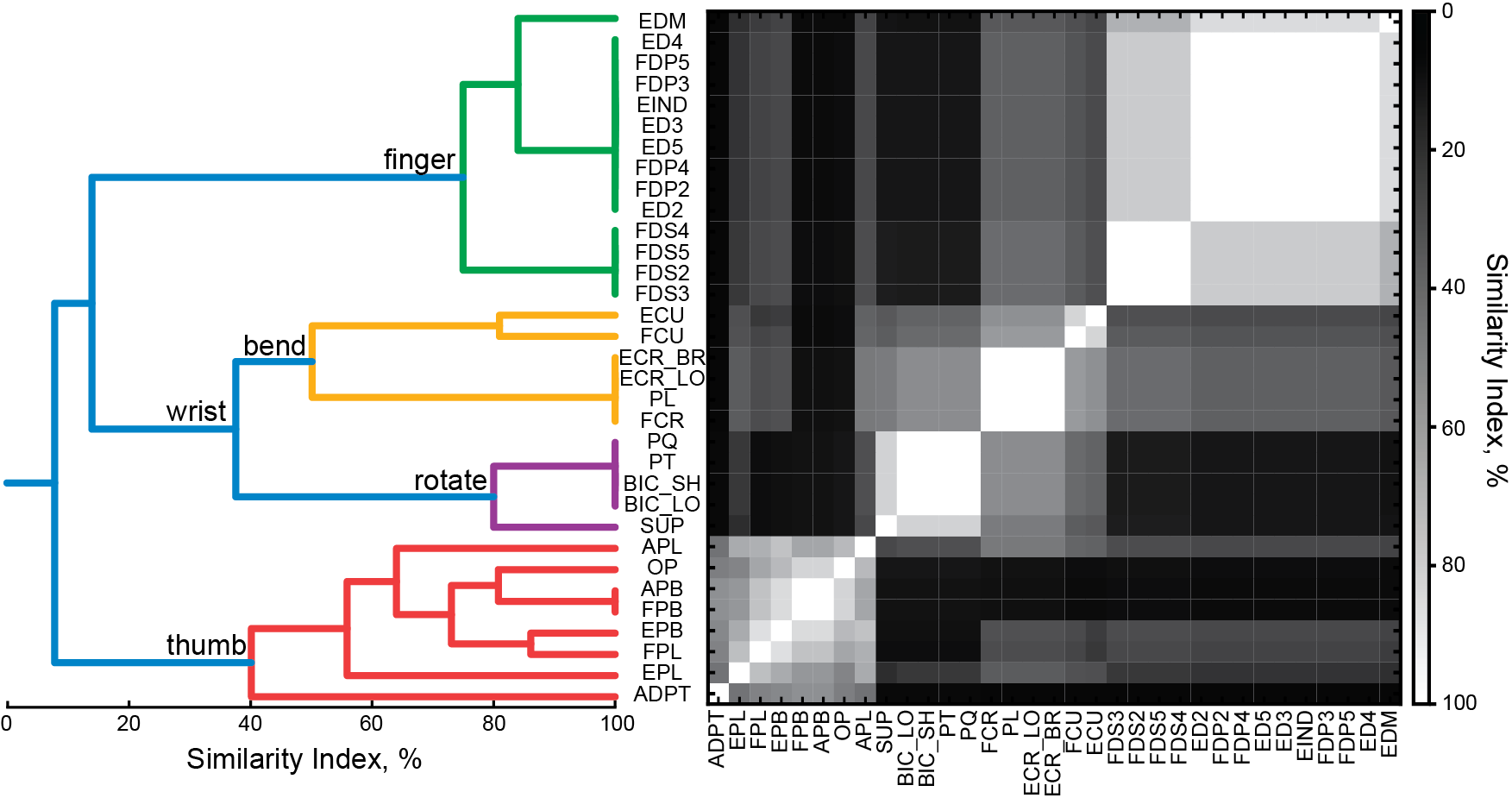


**S5 Figure**. Similarity of muscle structures using Similarity Index. Average-linkage dendrogram computed from the heatmap of pairwise Similarity Index. The distance between clusters was calculated as an average distance between elements of two clusters.
